# Memory Retrieval in Online Sentence Parsing: Empirical Evidence, Computational Modelling, and Simulations

**DOI:** 10.1007/s42113-024-00206-8

**Published:** 2024-07-01

**Authors:** Hiroki Fujita

**Affiliations:** Department of Linguistics, University of Potsdam, Karl-Liebknecht-Straße 24–25, 14476 Potsdam, Germany

**Keywords:** Memory retrieval, Revision, Quantifier float, Sentence parsing, Sentence processing, Language comprehension, Computational modelling, Computational simulations, ACT–R

## Abstract

This paper reports two experiments (Experiments 1 and 2) and computational simulations designed to investigate and model memory retrieval processes during real-time sentence processing. Central to this study is the hypothesis that linguistic information serves as a cue to retrieve target representations from memory during dependency formation. The basis for this cue-based memory retrieval stems from research showing that non-target representations that match a set of retrieval cues interfere with target retrieval. The susceptibility to this similarity-based interference has been debated in the sentence processing literature, and various hypotheses and models have been formulated and developed. This issue is addressed empirically in Experiments 1 and 2, which investigated similarity-based interference in sentences with a floating quantifier. Bayesian linear mixed models and Bayes factor analyses suggested similarity-based interference. However, the patterns of interference were not consistent with existing theories and models. To reconcile these findings within the framework of cue-based memory retrieval, this paper implements *the Revision Integrated Cue-Based* (RICB) model based on the ACT–R architecture. This model assumes that structural information is heavily weighted and incorporates the notions of initial retrieval and revision. The results of the simulations indicate that the RICB model successfully predicts the observed data, highlighting the central role of structural information and revision in memory retrieval during real-time sentence processing.

## Introduction

When we read a sentence, we often need to recover information about previously encountered words and link it to the information provided by the current word in order to understand the sentence. Our brains do this easily and quickly in real time when we simply scan the sentence. Unravelling the mechanisms that underlie this real-time language comprehension is a major challenge in cognitive science.

A standard assumption in language comprehension research is that sentences have underlying syntactic structures (Chomsky, 1957; Crocker, 1996; L. Frazier, 1979; Gibson, 1991; Kimball, 1973; Phillips, 1996; Sturt, 1997) from which meaning is derived, and that these syntactic structures are assigned incrementally during real-time processing. Under this assumption, relations are established between elements within the syntactic structures rather than between words in the sentence. These relations are often called *dependency relations* or simply *dependencies* (e.g., Chomsky, 1956; Fodor, 1978). Specifically, a dependency relation *R* on a set of elements *E* is defined as a subset of *E* × *E*. Within the syntactic structures of a sentence, two elements are in a dependency relation if one of the elements requires the presence of the other in the syntactic structures, or the properties of one of the elements vary due to the presence of the other. For example, consider the following sentences where the subject noun phrase (NP) and the auxiliary verb are in a dependency relation.

- [_NP1_ The sisters of [_NP2_ the girls]] [_T_ were] walking back home.
- [_NP1_ The sister of [_NP2_ the girls]] [_T_ were] walking back home. 

In (1a/b), the square brackets, labelled as NP1, NP2 and T, represent part of the syntactic structures underlying these sentences.^1^ NP1 is the sentence subject NP, and “were” is an auxiliary verb. According to the definition of dependency relations, the elements derived from “The sisters…” and “were” belong to a dependency relation, given their covariance in form.

Recent studies argue that the formation of dependency relations during real-time sentence processing requires memory retrieval. One phenomenon that may shed light on the characteristics of this memory retrieval process is *similarity-based interference*. Assuming that the properties of words, such as number and gender, are represented as features, e.g., “the sisters” as *NP*_[+*plural*]_ and “the sister” as *NP*_[+*singular*]_, similarity-based interference refers to interference due to feature similarity. For example, the sentence in (1b) is ungrammatical because of the mismatch in number between NP1 and the auxiliary verb (“The sister…were”). It is known that processing difficulties arise when the parser recognises the violation of such a linguistic agreement (*mismatch effects*; e.g., Cunnings & Felser, 2013; M. Frazier et al., 2015; Fujita, 2024; Fujita & Yoshida, 2024; Giskes & Kush, 2021; Jäger et al., 2020; Kazanina et al., 2007; Kim et al., 2020; Pearlmutter et al., 1999; Sturt, 2003; Sturt & Kwon, 2024; Vasishth et al., 2008; Wagers et al., 2009). Importantly, the sentence in (1b) contains another NP (“the girls”), which is unrelated to the subject-verb dependency relation and matches the auxiliary verb in number. Previous work suggests that this feature-matching plural *distractor* reduces the size of mismatch effects at the auxiliary verb compared to when the distractor is singular and thus does not match the verb in number, as in (2) below (e.g., Dillon et al., 2013; Fujita & Cunnings, 2023; Kim et al., 2020; Tucker et al., 2015; Wagers et al., 2009). These findings have been interpreted as evidence that at the second dependency element (“were”), the parser uses its lexical features (e.g., [+*plural*]) as retrieval cues to retrieve the first dependency element (NP1) from memory, a process known as *cue-based memory retrieval*.

(2) [_NP1_ The sister of [_NP2_ the girl]] [_T_ were] walking back home.

Similarity-based interference has been widely investigated in the context of cue-based memory retrieval. Despite this extensive research, several issues remain unresolved, in particular the conditions under which similarity-based interference occurs. For example, some studies argue that interference occurs in both grammatical and ungrammatical sentences (e.g., Vasishth & Engelmann, 2021), while others propose that it is restricted to ungrammatical sentences (e.g., Wagers et al., 2009). To date, a variety of cue-based hypotheses and models have been proposed and developed (e.g., Dillon, 2014; Dillon et al., 2013; Kush, 2013; Parker, 2019a; Parker & Phillips, 2017; Van Dyke & McElree, 2011; Vasishth et al., 2019; Vasishth & Engelmann, 2021; Wagers et al., 2009). Previous research has also suggested that distractor position might influence cue-based memory retrieval (e.g., Arnett & Wagers, 2017; Parker & An, 2018) and that similarity-based interference might be specific to certain types of dependencies, indicating potential variations in memory retrieval processes across different dependency types (e.g., Dillon et al., 2013; Orth et al., 2021).

Importantly, these unresolved issues are compounded by the claim that detecting similarity-based interference requires a large sample size, particularly in grammatical sentences, and that previous research has lacked sufficient statistical power. Experiments 1 and 2 were designed to address these issues. To overcome the statistical power limitation, each experiment included a substantial sample size as determined by a power analysis (640 participants and 24 item sets per experiment). Experiments 1 and 2 investigated similarity-based interference and the influence of distractor position by examining the processing of floating quantifiers (e.g., Koopman & Sportiche, 1991; Sportiche, 1988) and using relative clauses (e.g., Chomsky, 1965, 1977; de Vries, 2002; Kayne, 1994; Ross, 1967; Salzmann, 2019). Quantifier float, as exemplified in the sentence “John said that the girls recently all walked back home”, involves the separation of a quantifier (e.g., “all”) from its associate (e.g., “the girls”). According to the definition of dependency relations given earlier, these elements are in a dependency relation. The motivation for studying quantifier float stems from the lack of attention given to this dependency in the existing literature, even though its distributional properties provide an interesting test case for cue-based memory retrieval. Furthermore, there is currently conflicting evidence regarding the susceptibility to similarity-based interference in the quantifier float construction (Fujita & Cunnings, 2023). Therefore, the study of quantifier float potentially contributes to our understanding of the universality of similarity-based interference.

A brief overview of the results: Experiments 1 and 2 revealed the presence of mismatch effects, suggesting that memory retrieval during the real-time processing of floating quantifiers adheres to structural constraints. Similarity-based interference was observed in ungrammatical sentences, irrespective of the distractor position. However, there was no clear evidence for interference in grammatical sentences. To reconcile these findings within the framework of cue-based memory retrieval, a computational model was proposed and evaluated through simulations. The results of these simulations demonstrated that the proposed model successfully predicted the observed data.

### Theories of Cue-Based Memory Retrieval and Computational Models

**Fig. 1:**
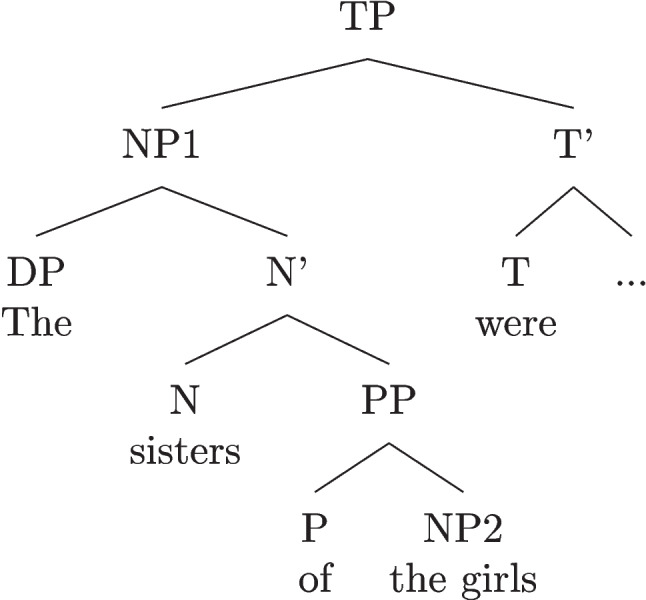
The hierarchical syntactic structures underlying part of the sentence in (1a)

In language, some words seem to be associated with other words in certain ways, and these associations seem to be determined or influenced by the position of the words in the sentence. Any theory and model of memory retrieval during sentence processing must integrate this distributional aspect of language to identify what serves as the target representation. This paper assumes that each word projects hierarchical structures, as shown in Fig. 1, which illustrates part of the hierarchical structures underlying the sentence in (1a). Dependency relations are determined on the basis of these hierarchical structures. In the grammar adopted in this paper (Chomsky, 1981), an auxiliary verb basically forms a dependency with an NP in the specifier of a TP. In Fig. 1, NP1, but not NP2, occupies this position. Consequently, in (1a), there is a dependency relation between NP1 and the auxiliary verb. Throughout the paper, underlying syntactic structures are often represented by labelled square brackets, as in (1a/b).^2^

Assuming that dependency relations are determined or influenced by the positions of the elements in the syntactic structures, the assignment of grammatical structures is a prerequisite for the formation of grammatical dependencies during real-time sentence processing. A large body of research has suggested that real-time language comprehension involves the assignment of grammatical syntactic structures (e.g., Aoshima et al., 2004; M. Frazier et al., 2015; Hall & Yoshida, 2021; Kazanina et al., 2007; Kush et al., 2017; Omaki & Schulz, 2011; Phillips, 2006; Schneider & Phillips, 2001; Yoshida et al., 2013, 2014). However, as discussed in the Introduction, previous studies have suggested that online dependency formation does not *strictly* adhere to structural constraints. Some of these studies claim to be consistent with theories of cue-based memory retrieval, which argue that the lexical features of the second dependency element (e.g., [+*plural*] from “were”) serve as retrieval cues (e.g., the number cue) to retrieve the first dependency element (e.g., NP1) from memory. These theories must postulate the existence of retrieval cues derived from the underlying syntactic structures (see Alcocer & Phillips, 2012; Kush, 2013).^3^

In the language comprehension literature, there are several models that incorporate cue-based memory retrieval. *The activation-based model* (Lewis & Vasishth, 2005; Vasishth & Engelmann, 2021), implemented on the basis of *the Adaptive Control of Thought–Rational* (*ACT–R*; Anderson, 2007; Anderson et al., 2004; Anderson & Lebiere, 1998) architecture, is one such model. In this model, each element is assumed to have an activation value. During memory retrieval, the element with the highest activation that reaches a retrieval threshold is retrieved. The higher the activation value, the faster the memory retrieval. In essence, the total activation (*T*) of a given element *i* is calculated by summing the values of the base-level activation (*B*), the spreading activation (*A*), and the stochastic noise (*N*). The calculation of these activation values is summarised in Table 1.

**Table 1:** Some crucial components of the activation-based model. *i* = element, *j* = cue

Component	Equation	Description
*B*_*i*_ (Base-level activation)	${B}_i={b}_i+\ln \left(\sum\limits_{k=1}^n{t_k}^{-d}\right)$	*t* = time *k* = retrieval *d* = decay parameter *b* = constant
*A*_*i*_ (Spreading activation)	${A}_i=\sum\limits_{j=1}^n{W}_j{S}_{ji}$	*W*_*j*_ = weight *S*_*ji*_ = associative strength between *j* and *i*
*N*_*i*_ (Noise)	$Normal\left(0,\sqrt{\frac{\pi^2}{3}{x}^2}\right)$	*x*^2^ = scaling parameter
*RT*_*i*_ (Retrieval time)	${RT}_i=F{e}^{-\left(f\bullet {T}_i\right)}$	*F*, *f* = scaling parameters

The base-level activation represents previous retrievals and time-dependent decay. Most important for the present study is the spreading activation. In the activation-based model, cue weights are assumed to be uniformly distributed across cues (i.e., 1/|*Cue*|). The associative strength is calculated as follows: *S*_*ji*_ = *m* +  *ln* (*P*(*i*| *j*)), where *m* is a constant and refers to the maximum strength of association that *i* can have. The rightmost term is a conditional probability that calculates the probability of *i* when *j* is present. A standard assumption in sentence processing research is that all *i* that match *j* are equally likely when *j* is present. Therefore, the conditional probability is simply the reciprocal of the number of *i* in memory that match *j*. If multiple *i* match *j*, the associative strength between each *i* and *j* decreases (*the fan effect*; Anderson, 1974). Lower activation leads to longer retrieval times because the retrieval time (*RT*) of *i* is calculated by the product of *F* and an exponential function with a negative exponent determined by the total activation.

Increased retrieval times due to the fan effect are referred to as *inhibitory interference* (Dillon et al., 2013). For example, the activation-based model predicts inhibitory interference in “(1a) [_NP1_ The sisters of [_NP2_ the girls]] were…” because NP1 and NP2 match the number cue, leading to a reduction in spreading activation to NP1 from this cue (*S*_*num*, *NP*1_ ≈ .31; feature match = 1, m = 1, num = number). This contrasts with sentences such as the one in (3) below, where NP2 does not match the number cue (“the girl…were”), and consequently, there is no reduction in spreading activation to NP1 from the number cue (*S*_*num*, *NP*1_ = 1; feature mismatch = 0).

(3) [_NP1_ The sisters of [_NP2_ the girl]] were walking back home.

As mentioned in the Introduction, a large body of research has demonstrated that the size of mismatch effects is attenuated in ungrammatical sentences, such as “(1b) [_NP1_ The sister of [_NP2_ the girls]] were…”, when the distractor (NP2) matches the auxiliary verb in number, compared to when it mismatches it, as in “(2) [_NP1_ The sister of [_NP2_ the girl]] were…”. In the activation-based model, this effect, called *facilitatory interference*, is predicted in the following scenario. In (1b), focusing on the structure-based and number-based cues, NP1 only matches the structure cue (*A*_*NP*1_ = .5; *W*_*j*_ = .5, *S*_*ji*_ = 1), and NP2 only matches the number cue (*A*_*NP*2_ = .5). Since there is no fan effect (*S*_*str*, *NP*1_ = *m* + ln(1), *S*_*num*, *NP*2_ = *m* + ln(1); str = structure), NP1 and NP2 receive the maximum activation from each cue. In (2), NP1 receives full activation from the structure-based cue (as in (1b)). However, NP2 receives no activation from either cue (*A*_*NP*2_ = 0) because it does not match them. Activation values fluctuate from trial to trial due to stochastic noise. Consequently, in (1b), either NP1 or NP2, if the retrieval threshold is reached, is retrieved with a probability of about 0.5, depending on the noise fluctuations, but the retrieved element consistently has the higher activation. In (2), NP1 is always retrieved (again if the retrieval threshold is reached), even when its activation is relatively low due to stochastic noise. As a result, over multiple trials, the activation-based model predicts shorter retrieval times in (1b) than in (2).

As shown in Table 1, the activation-based model assumes an additive combination of cues (i.e., $\sum_j{W}_j{S}_{ji}$). In contrast to the activation-based model, some studies have proposed that retrieval cues operate through a multiplicative combination (see Dillon et al., 2013; Kush, 2013; Parker, 2019a). In sentence processing research, this cue combination scheme (Clark & Gronlund, 1996; Gillund & Shiffrin, 1984; Raaijmakers & Shiffrin, 1981) is primarily motivated by the proposition that memory retrieval resists similarity-based interference during online language comprehension. To illustrate, consider the following equation, which calculates the retrieval probability (*RP*) of *i* based on its match with *j*: ${RP}_i=\frac{\prod_j{S}_{ji}^{W_j}}{\sum_i\prod_j{S}_{ji}^{W_j}}$. Assuming that feature match = 0.99 and feature mismatch = 0.01, this equation indicates that only partially matching elements make a minimal contribution relative to fully matching elements due to the interdependence of retrieval cues. For example, in sentence (1a) “[_NP1_ The sisters of [_NP2_ the girls]] were…”, if we assume *W* = 1, *RP*_*NP*1_ is .990. If the distractor does not match the auxiliary verb in number , as in “(3) [_NP1_ The sisters of [_NP2_ the girl]] were…”, it is .999. Thus, *RP*_*NP*1_ ≈ 1, irrespective of whether the distractor matches in number or not.

In the case of ungrammatical sentences, a similar retrieval probability is obtained for NP1 when the distractor does not match in number, as in (2) “[_NP1_ The sister of [_NP2_ the girl]] were…”. However, when the distractor matches in number, as in (1b) “[_NP1_ The sister of [_NP2_ the girls]] were…”, the denominator becomes twice the value of the numerator, resulting in a retrieval probability of 0.5. These probabilities provide a basis for assessing similarity-based interference in online language comprehension. The calculation of retrieval times can take a variety of forms, depending on assumptions about the cognitive processes involved in memory retrieval. One assumption, computationally consistent with proponents of multiplicative cue combination in language comprehension research, is that the parser searches for another element whenever no grammatical dependency is formed (Yadav et al., 2023).^4^

This search process will be referred to as *revision*.^5^ The need for revision is defined in terms of dependency relations. Let *D* = {*R*_1_, *R*_2_, …*R*_*n*_} be a set of dependency relations, and let *x* and *y* represent temporal points during sentence processing, where *x* < *y*. Then, revision is considered necessary at *y* if *D*_*x*_ ⊈ *D*_*y*_. According to this definition, we can assume that revision becomes necessary at the second dependency element *i*_*b*_ when a feature-mismatching element *i*_*a*_ is retrieved because, during the processing of *i*_*b*_, 〈*i*_*a*_, *i*_*b*_〉 ∈ *D* at *x*, but 〈*i*_*a*_, *i*_*b*_〉 ∉ *D* at *y* due to the violation of linguistic agreement. Since revision is an additional cognitive process, it takes some time to complete (see Cunnings & Fujita, 2021; Ferreira & Clifton, 1986; Ferreira & Henderson, 1991; Fodor & Ferreira, 1998; Frazier, 1979; Frazier & Rayner, 1982; Fujita, 2021b; Fujita, 2023; Fujita & Cunnings, 2020, 2021a, 2021b; Gibson, 1991; Pritchett, 1992; Sturt, 1997; Sturt et al., 1999). In other words, assuming that retrieval times follow a normal distribution, *RT*_*i*_~*Normal*(*μ*, *σ*), retrieval times with revision can be expressed as: *RT*_*i*_~*Normal*(*μ* + *α*, *σ*).

In grammatical sentences, revision occurs with a probability of 1 – *RP*_*NP*1_. As explained earlier, the retrieval probability of NP1 in grammatical sentences remains close to 1 regardless of the distractor features. Therefore, retrieval times almost always follow the *Normal*(*μ*, *σ*) distribution, resulting in minimal interference effects. In ungrammatical sentences, revision always occurs because no element fully matches the retrieval cues. Therefore, although the retrieval probability of NP1 varies depending on the presence or absence of a feature-matching distractor, retrieval times always follow *Normal*(*μ* + *α*, *σ*). As a result, the multiplicative cue-based model predicts processing costs due to mismatch effects, but no interference in both grammatical and ungrammatical sentences.

### Conditions on Similarity-Based Interference

There is an ongoing debate about the factors that may influence memory retrieval. One area of discussion focuses on a so-called *grammatical asymmetry*, where interference effects are observed in ungrammatical sentences but not in grammatical sentences. Specifically, a large body of previous research has demonstrated that online memory retrieval in English is susceptible to facilitatory interference (e.g., Cunnings & Sturt, 2018; Fujita & Cunnings, 2022, 2023; Fujita & Yoshida, 2024; González Alonso et al., 2021; Jäger et al., 2020; Kim et al., 2019, 2020; Lago et al., 2015; Wagers et al., 2009). In contrast, the evidence regarding inhibitory interference remains inconclusive. While many studies have found no inhibitory interference (e.g., Cunnings & Fujita, 2023; Dillon et al., 2013; Fujita & Cunnings, 2022, 2023; Fujita & Yoshida, 2024; González Alonso et al., 2021; Jäger et al., 2020; Lago et al., 2015; Wagers et al., 2009), some have reported its presence (Patil et al., 2016; Van Dyke, 2007; Van Dyke & McElree, 2011) or observed interference effects against it (Cunnings & Felser, 2013; Sturt, 2003). The presence of inhibitory and facilitatory interference is consistent with the activation-based model, whereas the absence of inhibitory interference is consistent with the multiplicative cue-based model.

The grammatical asymmetry has led to the formulation of several hypotheses about memory retrieval. For example, Wagers et al. (2009) argue that cue-based memory retrieval occurs only when a prediction conflicts with the actual input. This retrieval process leads to similarity-based interference only in ungrammatical sentences.

To illustrate Wagers et al.’s argument, consider the subject-verb agreement in (1a) “[_NP1_ The sisters of [_NP2_ the girls]] [_T_ were]….”. When encountering NP1, the parser predicts a verb marked with a plural feature (e.g., “were”) and maintains information about it (Gibson, 1998; Kim et al., 2020). In this context, it is possible to predict the presence of a verb because it is a component of the obligatory structure; the sentence becomes ungrammatical in its absence (see Abney, 1989; Aoshima et al., 2004; L. Frazier & Fodor, 1978; Fujita, 2023; Gibson, 1991, 1998; Gorrell, 1995; Pritchett, 1992; Weinberg, 1999; Yoshida et al., 2013). As the auxiliary verb “were” materialises, it corresponds to the predicted representation in terms of number and grammatical categories. Consequently, the parser integrates it into the current structure and forms the subject-verb dependency without cue-based memory retrieval being triggered.

In the case of the ungrammatical sentence (1b) “[_NP1_ The sister of [_NP2_ the girls]] [_T_ were]….”, the parser predicts a singular verb (e.g., “was”). However, the actual verb “were” is inconsistent with this prediction in terms of number. According to Wagers et al., mismatch effects occur in this case, and the parser then attempts to check the properties of NP1 by retrieving its information from memory, using the features of the verb as retrieval cues. This process sometimes leads to misretrieval of the distractor, resulting in facilitatory interference.

Fujita and Yoshida (2024) also argue for the grammatical asymmetry, but suggest that inhibitory interference does not occur because similarity-based interference is a phenomenon of last resort. Specifically, they claim that in ungrammatical sentences, sentence processing is susceptible to facilitatory interference because, after mismatch effects occur, the parser searches for an element that matches the retrieval cues in terms of lexical features for interpretive reasons. In grammatical sentences, the parser does not need to form such a structurally impermissible dependency, because there is always a grammatical dependency relation.

Although, as discussed above, many studies have observed facilitatory interference, there are a few that have suggested its absence. For example, Dillon et al. (2013) observed facilitatory interference in subject-verb agreement, but not in *reflexive resolution*, as illustrated in (4a/b) below.

- [_TP_ [_NP1_ The bodybuilder who saw [_NP2_ the trainers]] injured themselves last night].
- [_TP_ [_NP1_ The bodybuilder who saw [_NP2_ the trainer]] injured themselves last night].

The sentences in (4a/b) contain the reflexive pronoun “themselves”, which is referentially dependent on another NP, its *antecedent*. This reference relation is subject to structural constraints. Informally and approximately, a reflexive takes a locally c-commanding NP as its antecedent (Chomsky, 1981). C-command refers to a structural relation between nodes in a tree as in Fig. 1 and is defined as follows: node *a* c-commands node *b* if (i) node *c* is a sister of *a* and (ii) *b* = *c* or *c* dominates *b* (Reinhart, 1976).^6^ In (4a/b), NP1 c-commands the reflexive, but NP2 does not. Thus, NP1 is a structurally accessible antecedent and NP2 is a distractor. A reflexive must agree with its antecedent NP in number. In (4a), only NP2 matches the reflexive in number, and in (4b), both NP1 and NP2 do not match. The activation-based model predicts facilitatory interference in (4a) compared to (4b), in contrast to the findings of Dillon et al. On the other hand, the multiplicative cue-based model predicts no facilitatory interference, in line with Dillon et al. The results of Dillon et al. could indicate that different cue combination mechanisms are used for different types of dependencies. However, recent studies with large sample sizes have reported facilitatory interference in reflexive resolution, suggesting that online reflexive resolution in ungrammatical sentences is susceptible to interference effects, at least under certain circumstances (Fujita & Yoshida, 2024; Jäger et al., 2020).

There is also an ongoing debate about whether distractor position affects memory retrieval (Arnett, 2016; Arnett & Wagers, 2017; Engelmann et al., 2019; Fujita & Yoshida, 2024; Parker & An, 2018; Van Dyke & McElree, 2011). Parker and An (2018) reported that memory retrieval is insusceptible to similarity-based interference in subject-verb dependencies when the distractor is in a subject or object position, as in [_NP1_ *The boys who saw [*_*NP2*_ *the girls]] were walking back home*. In this sentence, NP2, the distractor, is a direct object of “saw”. In this paper, the term *subject position* refers to the specifier of a tensed TP, and the term *object position* refers to the complement of a verb. According to Parker and An, subjects and objects are the primary content of sentences and are therefore encoded distinctively; as a result, the parser can easily identify a distractor in a subject or object position as unrelated to the subject-verb dependency, leading to no interference (but see Fujita & Yoshida, 2024).

Some studies suggest different roles for subject and object positions (Arnett, 2016; Arnett & Wagers, 2017; Engelmann et al., 2019). Engelmann et al. (2019) argue that a distractor in a subject position is to some extent prominently encoded, leading to increased interference. Similarly, Arnett and Wagers (2017) argue for the role of subject positions in interference, but their argument is rooted in linguistic theory. As noted earlier, a finite verb basically forms a dependency relation with an NP in the specifier of a tensed TP. Arnett and Wagers suggest, on the basis of their empirical findings, that the parser uses this information as a retrieval cue in subject-verb dependencies. Therefore, according to Arnett and Wagers, (increased) interference effects are expected from a distractor in the specifier of a finite TP when memory retrieval targets such a position.

## The Present Study

This paper first reports two experiments (Experiments 1 and 2) designed to investigate similarity-based interference. A computational model and simulations are presented in the General Discussion. Experiments 1 and 2 tested sentences with a *floating quantifier*, as shown in (5) below.

(5) [_TP_ [_NP1_ The boys who [_NP2_ the girls]] saw all walked back home from school].

**Fig. 2:**
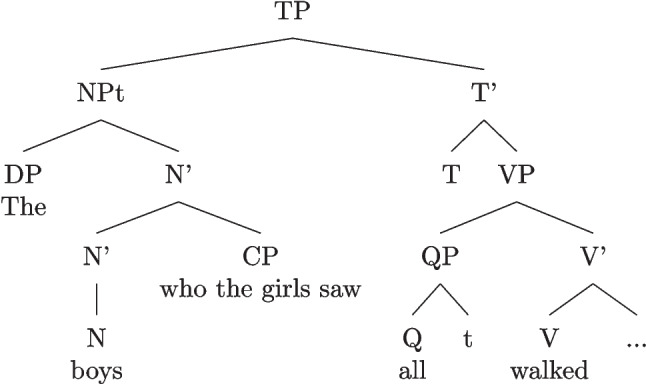
The hierarchical syntactic structure underlying part of the sentence in (5)

In English, universal quantifiers such as “all” usually appear immediately before their associates like “all the boys”. In (5), however, the quantifier “all” appears without an immediate NP. One possible analysis of this floating quantifier phenomenon is that the quantifier and its associate form a constituent, but at a particular stage of the derivation, only the associate moves to a higher position, leaving the quantifier stranded in a lower position (e.g., Al Khalaf, 2019; Giusti, 1990; McCloskey, 2000; Sportiche, 1988; Zyman, 2018). Figure 2 provides a visual illustration of this analysis. In (5), although the quantifier is preceded by two plural nominals (NP1 and NP2), it can only form a dependency relation with NP1. This can be explained by the observation that in English, floating quantifiers are locally c-commanded by their associates, and NP1, but not NP2, c-commands the floating quantifier (see Fig. 2). Also, a floating quantifier must modify a plural nominal. For example, if NP1 in (5) is singular, the sentence becomes ungrammatical (“The boy who the girls saw all walked…”). These constraints provide a test case for similarity-based interference.

In the language comprehension literature, there is only one study that has examined similarity-based interference in the quantifier float construction, and the results are controversial with respect to online sentence processing (Fujita & Cunnings, 2023). Specifically, Fujita and Cunnings (2023) observed facilitatory interference in one reading experiment, but found no interference effects in another reading experiment, when the distractor was in the subject position of a relative clause, as in (5). Therefore, investigating quantifier float has theoretical importance in terms of understanding the universality of similarity-based interference and cue combination mechanisms (Dillon, 2014; Dillon et al., 2013; Orth et al., 2021; Parker, 2019a). In addition, quantifier float provides an intriguing test case for exploring cue-based memory retrieval for several reasons. First, the parser is unlikely to predict the presence of a floating quantifier in sentences such as (5). This is because the quantifier is not obligatory in the sentence structure, and the sentence lacks a contextual bias towards its presence (Fujita, 2023; Gibson, 1991; Gorrell, 1995; Weinberg, 1999). Consequently, the appearance of a floating quantifier should be unexpected, which may trigger cue-based memory retrieval to search for its associate, leading to inhibitory interference (Wagers et al., 2009). Second, the associate of a floating quantifier is not restricted to a subject position; it can also occupy an object position, as observed in sentences such as *John likes them all*. Given this distributional property, it is possible that the retrieval of the associate of a floating quantifier does not exclusively target subject positions. Thus, if we adopt the perspective suggested by Arnett and Wagers (2017), similar interference effects would be expected between subject and object positions. However, if we follow Engelmann et al. (2019), who argue that any element in a subject position is to some extent prominently encoded, we can expect increased interference effects from subject positions relative to object positions. It is also conceivable that real-time sentence processing is impervious to similarity-based interference when the distractor occupies a subject or object position (Parker & An, 2018). By investigating online dependency formation between floating quantifiers and their associates, the present study aims to provide insight into these theoretical issues. The influence of distractor position is assessed by placing a distractor in either a subject position (Experiment 1) or an object position (Experiment 2).

**Fig. 3:**
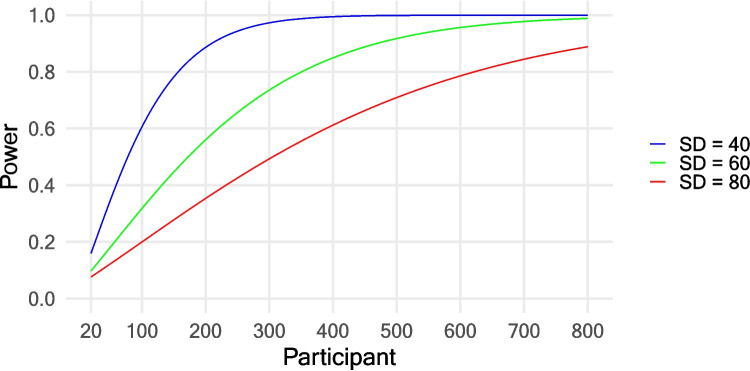
Power functions for inhibitory interference. The effect size (9ms) is based on Nicenboim et al. (2018)

An important consideration when investigating similarity-based interference is that its size may be small, particularly for inhibitory interference. For example, Nicenboim et al. (2018) reported an effect size of approximately 9 ms for inhibitory interference, with a 95% range of [0, 18] ms, based on pooled data from their two experiments. Assuming that this estimate approximates the true effect size, a simple power analysis suggests that, with a reasonable standard deviation (e.g., Parker, 2019a), 640 participants would be required to achieve an 80% probability of detecting inhibitory interference (see Fig. 3).

To ensure robust and reliable evidence of similarity-based interference, the present study included 640 participants and 24 sets of experimental sentences in the data analysis for each experiment.

## Experiment 1

Experiment 1 investigated similarity-based interference in the quantifier float construction with a distractor in the subject position. The experimental sentences, as shown in (6a–d) below, were taken from Fujita and Cunnings (2023).

- **Grammatical, Distractor match**
  The boys who saw the girls quite recently, all walked back home from school.
- **Grammatical, Distractor mismatch**
  The boys who saw the girl quite recently, all walked back home from school.
- **Ungrammatical, Distractor match**
  The boy who saw the girls quite recently, all walked back home from school.
- **Ungrammatical, Distractor mismatch**
  The boy who saw the girl quite recently, all walked back home from school.

In (6a/b), the floating quantifier “all” and its associate “The boy(s)…” match in number, whereas in (6c/d), they do not. In these sentences, the distractor “the girl(s)” occupies the subject position of a relative clause. This distractor matches the floating quantifier in number in (6a/c), but does not match it in (6b/d). If memory retrieval for floating quantifiers is a structure-dependent process, reading times at the quantifier should be longer in the ungrammatical conditions (6a/b) than in the grammatical conditions (6c/d) due to mismatch effects (Fujita & Cunnings, 2023; Wagers et al., 2009).

With respect to similarity-based interference, the activation-based model predicts longer reading times at the quantifier in (6a) than in (6b) due to inhibitory interference and shorter reading times at the quantifier in (6c) than in (6d) due to facilitatory interference (Lewis & Vasishth, 2005; Vasishth & Engelmann, 2021). In contrast, the multiplicative cue-based model predicts no similarity-based interference. The absence of similarity-based interference is also predicted by Parker and An (2018), who argue that similarity-based interference does not occur when a distractor is in a subject or object position.

### Participants

A total of 640 native English speakers were included in the data analysis. These participants were recruited via Prolific (https://prolific.co/). They had a university degree, were British citizens, and had lived in the UK for most of their lives before the age of 18.

### Materials

Experiment 1 included 24 sets of experimental sentences, as exemplified in (6a–d). In addition, the experiment included 72 filler sentences with a variety of syntactic structures. The critical region in all experimental sentences contained the quantifier “all”.

### Procedure

Reading times for each word were measured using a lexicality maze task implemented in PCIbex Farm (Zehr & Schwarz, 2018). In this task, participants were presented with each sentence word by word, along with a pseudoword, and had to press the button corresponding to the correct word that continued the sentence. If a pseudoword was selected, the trial was immediately terminated, accompanied by the display of a warning message, and the next trial began. The experimental file was generated using code available online (Fujita, 2021a). The experiment began with four practice trials, followed by 24 experimental sentences and 72 fillers presented in a pseudo-random order.

### Data Analysis

Reading times at the critical region (“all”) and the spillover region (“walked”) were analysed in Bayesian linear mixed models (Gelman et al., 2013; Lee & Wagenmakers, 2014; Nicenboim et al., 2023; Vasishth, 2023; Vasishth et al., 2023) with a log-normal likelihood, using the brms package (Bürkner, 2017) in R (R Core Team, 2022). In Bayesian statistics, the prior uncertainty is computed as a probability distribution, and the observed data update this uncertainty to compute the posterior distribution. The parameter values of research interest were sampled using Markov Chain Monte Carlo techniques.

Before data analysis, reading times below 100 milliseconds or above 10,000 milliseconds were excluded from the data, as these data points are likely to indicate lapses in attention. This procedure follows Fujita and Cunnings (2023). The models included the fixed effects of grammaticality (categorised as grammatical or ungrammatical), distractor (categorised as distractor match or distractor mismatch) and their interaction. The two levels of grammaticality and distractor were coded as 0.5/–0.5. The models included varying intercepts and slopes for participants and materials.

Regularising weakly informative priors were specified for the prior uncertainty. Specifically, *Normal*(6.5, 1) was specified for the intercept, and *Normal*(0, 1) for the other parameters. The prior distributions for the random effect standard deviations for participants and materials were truncated at 0. Each Bayesian linear mixed model involved four chains, each running for 4,000 iterations, with the first 2,000 iterations discarded as warm-up samples. Convergence was assessed using both R-hat diagnostics and visual inspection. All models reported in this paper converged.

In the results section, the estimates of the effects of theoretical interest are presented with their uncertainties, represented by the 2.5th percentile for the lower bound and the 97.5th percentile for the upper bound of the posterior distributions. This credible interval is denoted as 95% Crl [2.5th percentile, 97.5th percentile]. The posterior distributions are presented on the millisecond scale rather than on the logarithmic scale.

In addition to the 95% credible intervals, Bayes factor analyses were conducted for the interaction effects and similarity-based interference effects. A Bayes factor quantifies the relative evidence between two models by comparing their marginal likelihoods, which represent the probability of observing the data given the model specifications. Specifically, a Bayes factor (*BF*_10_) is calculated as follows: ${BF}_{10}=\frac{P\left( Data|{Model}_1\right)}{P\left( Data|{Model}_0\right)}$, where *Model*_0_ represents the null hypothesis, e.g., the interaction effect is zero (the model does not contain the interaction term), and *Model*_1_ represents an alternative hypothesis, e.g., the interaction effect is not zero (the model contains the interaction term). For example, *BF*_10_ = 3 suggests that the observed data are three times more likely to have occurred under *Model*_1_ than under *Model*_0_, providing evidence for *Model*_1_ and against *Model*_0_. In other words, ${BF}_{01}=\frac{1}{3}$ (i.e., ${BF}_{10}={\frac{1}{BF}}_{01}$).

The strength of evidence was interpreted according to the guideline proposed by Jeffreys (1961), as cited in Lee & Wagenmakers, 2014). This guideline, presented in Table 2 (Lee & Wagenmakers, 2014, p. 105), was considered as an approximate description of the strength, in line with the recommendation of Lee and Wagenmakers (2014).

**Table 2:** The interpretation of the Bayes factor. This guideline is proposed by Jeffreys (1961), as cited in Lee & Wagenmakers, 2014)

Bayes factor	Interpretation (*BF*_10_)
> 100	Extreme evidence for Model 1
30 – 100	Very strong evidence for Model 1
10 – 30	Strong evidence for Model 1
3 – 10	Moderate evidence for Model 1
1 – 3	Anecdotal evidence for Model 1
1	No evidence for Models 1 and 0
1/3 – 1	Anecdotal evidence for Model 0
1/10 – 1/3	Moderate evidence for Model 0
1/30 – 1/10	Strong evidence for Model 0
1/100 – 1/30	Very strong evidence for Model 0
< 1/100	Extreme evidence for Model 0

Bayes factors are sensitive to the prior distribution of parameters. For example, the weakly informative prior *Normal*(0, 1) assumed for the log scale fixed effects in this study gives a strong bias towards the null hypothesis. Therefore, more informative priors, namely, *Normal*(0, 0.025) and *Normal*(0, 0.05), were used for the Bayes factor analyses.

To calculate Bayes factors, models with varying intercepts for participants and materials were fitted using four chains and 22,000 iterations. The first 2,000 iterations of each chain were discarded and marginal likelihoods were calculated using the bridgesampling package (Gronau et al., 2020).

## Results

**Fig. 4:**
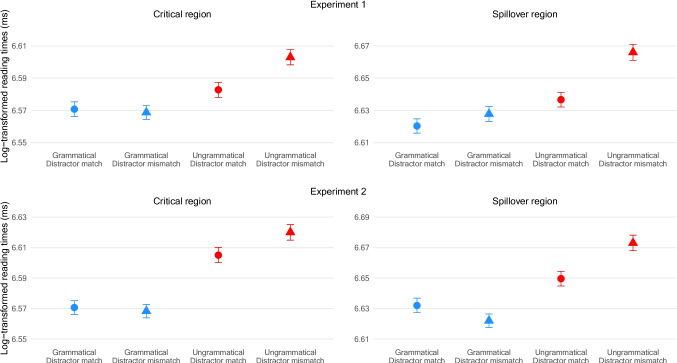
Log-transformed reading times at the critical (“all”) and spillover (“walked”) regions in Experiments 1 and 2 (“The boy(s) who the girl(s) saw/saw the girl(s) quite recently, all walked back home from school”). Error bars are standard errors

**Fig. 5:**
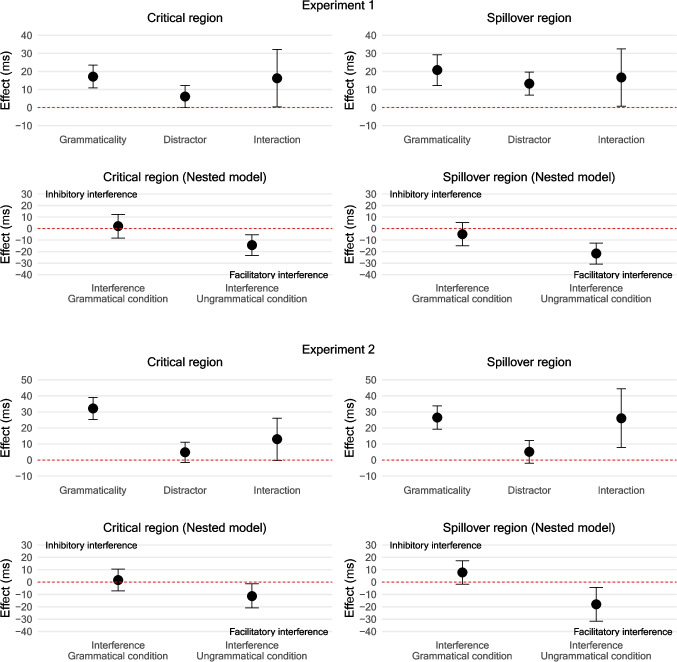
Estimates of the fixed effects and 95% credible intervals at the critical (“all”) and spillover (“walked”) regions in Experiments 1 and 2 (“The boy(s) who the girl(s) saw/saw the girl(s) quite recently, all walked back home from school”). For nested models, positive values on the y-axis indicate a reading time pattern consistent with inhibitory interference, while negative values indicate a reading time pattern consistent with facilitatory interference

**Fig. 6:**
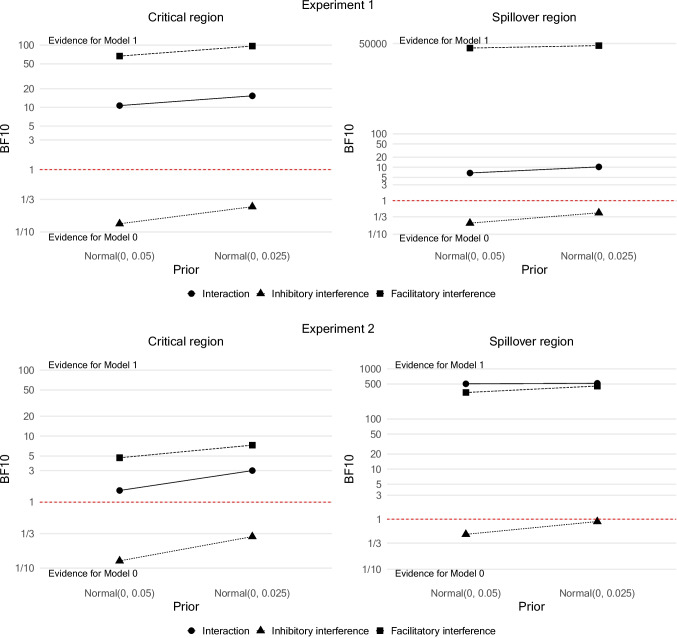
The results of the Bayes factor analyses at the critical (“all”) and spillover (“walked”) regions in Experiments 1 and 2 (“The boy(s) who the girl(s) saw/saw the girl(s) quite recently, all walked back home from school”)

The log-transformed reading times at the critical and spillover regions are illustrated in Fig. 4. Figure 5 displays the estimates of grammaticality, distractor, and their interaction, accompanied by 95% credible intervals. Figure 6 presents the results of the Bayes factor analyses. In cases where there was a sign of the interaction, a follow-up analysis was performed by fitting a nested model with a sum-coded fixed effect of distractor within each level of grammaticality.

### Critical Region

The effects of theoretical interest are grammaticality and its interaction with distractor. At the critical region, both effects show a positive sign. The 95% Crl for the grammaticality effect is [11, 24] ms, indicating longer reading times in the ungrammatical conditions than in the grammatical conditions (i.e., mismatch effects). The 95% Crl for the interaction effect is [0, 32] ms, and, as shown in Fig. 6, the Bayes factor analysis provides strong evidence for this effect.

For ungrammatical sentences, the nested model shows a negative sign of the distractor effect, with the 95% Crl [–23, –5] ms, indicating shorter reading times in the distractor match condition than in the distractor mismatch condition. This negative sign is consistent with facilitatory interference and is supported by the Bayes factor analysis, which provides very strong evidence for its presence. For grammatical sentences, the 95% Crl for the distractor effect is centred around zero ([–8, 12] ms), and the Bayes factor analysis provides moderate evidence against its presence, indicating no inhibitory interference.

### Spillover Region

The results at the spillover region closely mirror those at the critical region. The 95% Crl for the grammaticality effect is [12, 29] ms, indicating mismatch effects. There is also moderate to strong evidence for the interaction effect (the 95% Crl [1, 33] ms).

For ungrammatical sentences, the nested model shows shorter reading times in the distractor match condition than in the distractor mismatch condition (95% Crl [–31, –13] ms), a pattern consistent with facilitatory interference. The Bayes factor analysis provides extremely strong evidence for this effect. For grammatical sentences, there is a negative sign, with the effect being centred around zero, a pattern inconsistent with inhibitory interference (95% Crl [–15, 5] ms). The Bayes factor analysis suggests that the data are more likely to have been generated under the null hypothesis.

## Discussion

The mismatch effects observed at the critical and spillover regions indicate that when the floating quantifier appears, the parser attempts to retrieve its associate in a structurally permissible position. This finding suggests that memory retrieval during online language comprehension adheres to structural constraints. Importantly, the results provided evidence of facilitatory interference. However, there was no evidence of inhibitory interference. This pattern of interference is inconsistent with both the activation-based model (Vasishth & Engelmann, 2021) and the multiplicative cue-based model, but consistent with previous research observing the grammatical asymmetry. The results are also inconsistent with Parker and An (2018), who argue that similarity-based interference does not arise from a distractor in the subject position.

Experiment 2 further investigated similarity-based interference in the quantifier float construction, but with the distractor in an object position.

## Experiment 2

Experiment 2 used experimental sentences similar to those used in Experiment 1, but with the distractor in an object position, as shown in (7a–d) below.

- **Grammatical, Distractor match**
  The boys who saw the girls quite recently, all walked back home from school.
- **Grammatical, Distractor mismatch**
  The boys who saw the girl quite recently, all walked back home from school.
- **Ungrammatical, Distractor match**
  The boy who saw the girls quite recently, all walked back home from school.
- **Ungrammatical, Distractor mismatch**
  The boy who saw the girl quite recently, all walked back home from school.

The predictions for (7a–d) are analogous to those for (6a–d). First, if memory retrieval is a structure-dependent process, number mismatch effects should be observed at the quantifier, with longer reading times in the ungrammatical conditions (7c/d) than in the grammatical conditions (7a/b). Second, the activation-based model predicts inhibitory interference in (7a) compared to (7b) and facilitatory interference in (7c) compared to (7d). In contrast, the multiplicative cue-based model predicts no interference effects. Third, according to Parker and An (2018), interference effects should be absent in both (7a) and (7c) because the distractor is in the object position.

Regarding the potentially different roles of subject and object positions in memory retrieval, Engelmann et al. (2019) suggest that larger interference effects arise from subject positions relative to object positions. In contrast, Arnett and Wagers (2017) argue that such effects are limited to cases where memory retrieval targets subject positions. Given that the associates of floating quantifiers are not restricted to subject positions, these studies predict either that (7a–d) should elicit smaller interference effects than (6a–d), or that similar interference effects should occur between (7a–d) and (6a–d).

### Participants

Experiment 2 involved 640 native English speakers who had not participated in Experiment 1. These participants were recruited via Prolific from the same pool of participants as in Experiment 1.

### Materials

The materials were identical to those used in Experiment 1, the only difference being the use of subject relative clauses instead of object relative clauses.

### Procedure and Data Analysis

The procedure and data analysis were identical to those of Experiment 1.

## Results

The log-transformed reading times at the critical and spillover regions are presented in Fig. 4. The estimates of the fixed effects are displayed In Fig. 5 and the results of the Bayes factor analyses are presented in Fig. 6.

### Critical Region

The 95% Crl for the grammaticality effect is [25, 39] ms, indicating mismatch effects. There is also a sign of the interaction effect [0, 26] ms. The Bayes factor analysis suggests that the evidence for this interaction effect is weak to moderate.

For ungrammatical sentences, the nested model shows a negative sign (95% Crl [–21, –1] ms), a pattern consistent with facilitatory interference. The Bayes factor analysis provides moderate to strong evidence for this effect. For grammatical sentences, the effect is centred around zero (95% Crl [–7, 11] ms), and there is moderate to strong evidence for the null model. These analyses suggest the absence of inhibitory interference.

### Spillover Region

The results at the spillover region are similar to those at the critical region. The estimate of the grammaticality effect shows a positive sign (95% Crl [19, 34] ms), suggesting mismatch effects. The 95% Crl for the interaction effect is [8, 44] ms. The Bayes factor analysis provides the extremely strong evidence for the interaction effect.

The nested model shows that for ungrammatical sentences, reading times are shorter in the distractor match condition than in the distractor mismatch condition (95% Crl [–38, –4] ms), indicating facilitatory interference. The Bayes factor analysis provides extremely strong evidence for its presence. For grammatical sentences, there is a very weak sign of inhibitory interference (95% Crl [–2, 17] ms), but the Bayes factor analysis shows either no evidence for this effect or weak evidence against it.

### Analysis of the Pooled Data

**Fig. 7:**
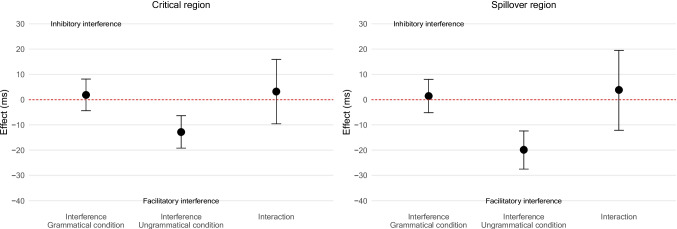
Estimates of the fixed effects and 95% credible intervals in the analysis of the pooled data. Interaction refers to an interaction effect between facilitatory interference and the experiment effect. Positive values on the y-axis indicate a reading time pattern consistent with inhibitory interference, while negative values indicate a reading time pattern consistent with facilitatory interference

**Fig. 8:**
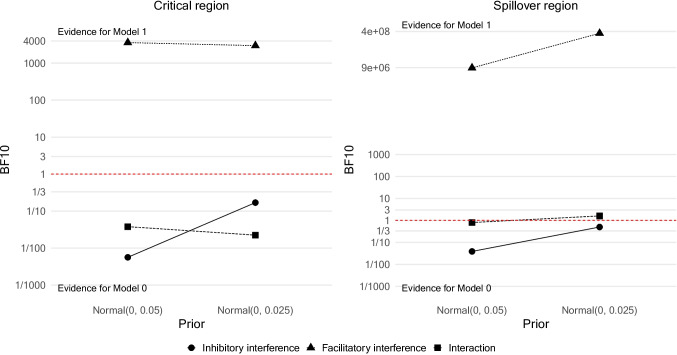
Bayes factors in the analysis of the pooled data. Interaction refers to an interaction effect between facilitatory interference and the experiment effect

Similar results were obtained in Experiments 1 and 2. To explore possible quantitative differences between these experiments, particularly regarding the influence of distractor position on facilitatory interference, an additional analysis was performed. In this analysis, the data from Experiments 1 and 2 were combined to compare reading times between the two experiments. Pooling the data also maximises statistical power, a critical consideration when investigating the susceptibility to inhibitory interference, as its effect size is potentially very small. Models for this analysis included fixed effects of grammaticality, inhibitory interference, and facilitatory interference, as well as a fixed effect of experiment (categorised as Experiment 1 or Experiment 2) and interaction effects. Experiment was a between-participants and between-materials factor. The models were fitted in a similar way to Experiments 1 and 2. In terms of the interaction effects, this analysis focuses on facilitatory interference, as this was the only interference effect for which Experiments 1 and 2 provided compelling evidence. The estimates of the fixed effects of theoretical interest and the results of the Bayes factor analyses are presented in Figs. 7 and 8, respectively.

For the critical region, there is a sign of facilitatory interference (the 95% Crl [–19, –6] ms), and the Bayes factor analysis provides extremely strong evidence for this effect. The 95% Crl for the interaction effect between facilitatory interference and the experiment effect is centred around zero (the 95% Crl [–10, 16] ms), and there is strong evidence against its presence. These analyses suggest similar facilitatory interference effects between Experiments 1 and 2. The 95% Crl for inhibitory interference is [–4, 8] ms, and there is moderate to extremely strong evidence against its presence.

For the spillover region, the Bayesian analysis shows a sign of facilitatory interference (the 95% Crl [–28, –12] ms), and the Bayes factor analysis provides extremely strong evidence for this effect. The interaction effect between facilitatory interference and the experiment effect is centred around zero (the 95% Crl [–12, 19] ms), and there is no evidence for this effect. There is also no indication of inhibitory interference (95% Crl [–5, 8] ms), and the Bayes factor provides moderate to strong evidence against its presence.

## Discussion

The results of Experiment 2 were analogous to those of Experiment 1. Mismatch effects were observed at the critical and spillover regions, suggesting that memory retrieval during online language comprehension is a structure-dependent process. There was also evidence of facilitatory interference, but the experiment found no evidence of inhibitory interference. Furthermore, the results indicated that distractor position had no discernible effect on memory retrieval. The analysis of the pooled data suggested a comparable size of facilitatory interference between Experiments 1 and 2.

## General Discussion

Experiments 1 and 2 investigated similarity-based interference in the quantifier float construction. The primary aim of these experiments was to address unresolved issues in language comprehension research before delving into computational modelling. These issues concern the susceptibility of online memory retrieval for floating quantifiers to similarity-based interference, the resistance to inhibitory interference, and the influence of distractor position. Resolving these issues is essential for the development of a computational model of memory retrieval processes during online language comprehension.

Clear mismatch effects were observed at the floating quantifier in both Experiments 1 and 2, suggesting that upon encountering a floating quantifier, the parser attempts to form a dependency with its associate in a structurally permissible position. Importantly, the results provided evidence for the susceptibility to facilitatory interference, but there was no evidence of inhibitory interference. The experiments also provided no clear evidence that distractor position influences online memory retrieval. In the following sections, the implications of these results are first discussed in detail. Then, a computational model based on this discussion is presented and evaluated through simulations.

### Similarity-Based Interference in Quantifier Float

As discussed in the Introduction, similarity-based interference has been extensively studied in the context of cue-based memory retrieval. However, little attention has been paid to floating quantifiers, and the susceptibility to similarity-based interference in the quantifier float construction remains controversial (Fujita & Cunnings, 2023). Fujita and Cunnings (2023) suggest that if the processing of floating quantifiers is impervious to similarity-based interference, it may be because quantifiers and their associates may form a constituent that functions as a cohesive unit. However, the present study suggested that online memory retrieval for floating quantifiers is susceptible to facilitatory interference in a manner similar to the formation of other dependency relations.

Facilitatory interference was observed despite the distractor occupying a subject (Experiment 1) or an object (Experiment 2) position. This finding poses a challenge to studies that argue that facilitatory interference does not arise from these positions (Parker & An, 2018). Recall that Parker and An base their argument on the premise that subjects and objects play a crucial role in sentence interpretation. They suggest that a distractor in a subject or object position is uniquely encoded, leading to no interference effects, because the parser can easily determine that it is not structurally permissible. However, some other studies have reported that interference effects arise from a distractor in a subject or object position (Fujita & Cunnings, 2023; Fujita & Yoshida, 2024; Sturt & Kwon, 2024; Tucker et al., 2015; Wagers et al., 2009). Given these studies, it is plausible to assume that subject or object positions alone do not nullify interference effects (see also Fujita & Yoshida, 2024, who suggest that subject or object positions might reduce interference effects when the distractor is in a finite clause).

The present study also found no evidence that a distractor in a subject position elicits greater interference effects than in an object position. There are two possible interpretations of this finding. The first concerns the distribution of the quantifier float construction. As mentioned in the Introduction, the associates of quantifiers are not restricted to subject positions. Therefore, it is conceivable that information specific to a subject position, such as the specifier of a TP and nominative case, is not used as a retrieval cue for accessing the associate of the quantifier in memory (Arnett & Wagers, 2017). Alternatively, subject position may lead to increased interference effects (Engelmann et al., 2019), but its influence is subtle and therefore difficult to detect. This interpretation stems from the very small numerical differences in facilitatory interference observed between Experiments 1 and 2. Further research is needed to disentangle these possibilities.

### Cue-Based Computational Models and Simulations

The evidence for facilitatory interference and against inhibitory interference challenges both the activation-based model, which predicts both types of interference (Lewis & Vasishth, 2005; Vasishth & Engelmann, 2021), and the multiplicative cue-based model, which predicts no interference (Dillon et al., 2013; Parker, 2019a). In contrast, Experiments 1 and 2 are consistent with a large body of previous research showing the grammatical asymmetry (e.g., Wagers et al., 2009). However, the results raise the question of why memory retrieval during online language comprehension may be susceptible to facilitatory interference but not to inhibitory interference.

As discussed in the Introduction, Wagers et al. (2009) propose, on the basis of empirical evidence from their study of subject-verb agreement, that the absence of inhibitory interference results from the parser’s selective engagement in cue-based memory retrieval. To reiterate, Wagers et al. argue that cue-based memory retrieval occurs when predictive parsing conflicts with the actual input. However, this proposal does not hold for all types of dependencies (Dillon et al., 2013; Fujita & Yoshida, 2024). I will discuss this issue in more detail below.

It is reasonable to assume that prediction influences the formation of subject-verb dependencies, where the verb serves as an obligatory component of the sentence structure (Fujita, 2023; Gibson, 1991, 1998; Weinberg, 1999). However, predictive parsing is not always an option during dependency formation. As discussed in the Introduction, it is unlikely that the parser predicts a floating quantifier during real-time sentence processing. The feasibility of predictive parsing in the quantifier float construction depends on contextual support. This scenario is also true for reflexives. The parser is unlikely to predict the presence of reflexives unless there is a strong contextual bias (Dillon et al., 2013; Fujita & Yoshida, 2024). Consequently, reflexives should also often be resolved in a bottom-up manner during real-time sentence processing, without any predicted representations.

According to the hypothesis proposed by Wagers et al. (2009), we can postulate the following parsing and memory retrieval processes in grammatical sentences with a floating quantifier. After the parser encounters a plural NP1 (“The boys who the girls…”), it assigns the corresponding hierarchical structure. The parser then predicts a plural verb for subject-verb agreement ([_TP_ [_NP1_ The boys who [_NP2_ the girls] saw] [_VP_ V_[+plural]_]]). However, the appearance of the floating quantifier contradicts this prediction because its grammatical category is not V. If this prediction error triggers cue-based memory retrieval to search for its associate for dependency formation, we can expect increased reading times at the quantifier, either due to inhibitory interference (Lewis & Vasishth, 2005) or due to the formation of the ungrammatical dependency, in contrast to the results of the present study.^7^

Fujita and Cunnings (2023) suggest that cue-based memory retrieval may be unnecessary in the grammatical sentences with a floating quantifier tested in their study because the floating quantifier corresponds to the predicted representation in terms of number (e.g., predicted representation: [_TP_ [_NP1_ The boys who [_NP2_ the girls] saw] [_VP_ V_[+plural]_]], input: all_[+plural]_). However, as noted in the Introduction, the associates of floating quantifiers are not restricted to the specifier position of a TP. Therefore, this proposal raises the question of how the parser determines that NP1 is the grammatical associate of the quantifier without memory retrieval.^8^

To predict the grammatical asymmetry within the framework of cue-based memory retrieval, two key assumptions can be made. The first assumption is that the structure-based cue is heavily weighted during initial memory retrieval. Initial memory retrieval is defined as the memory retrieval process triggered upon recognition of the input word. That is, given a sequence of memory retrievals within an input word, ${\left({M}_i\right)}_{i=1}^N$, where *M* refers to memory retrievals, *M*_1_ is the initial memory retrieval. The second assumption is that, during the revision process, the parser uses cue-based memory retrieval to form dependencies that do not adhere to structural constraints, but are consistent with lexical features. In other words, cue-based memory retrieval is invoked twice in ungrammatical sentences: once during the initial memory retrieval and once during the revision process. The memory retrieval during revision does not rely on the structure-based cue, because it is evident that it is not conducive to the formation of lexical feature-matching dependencies. This revision process may result from the parser’s attempt to find an element that licenses linguistic agreement in terms of lexical features, which can further be attributed to interpretive reasons (Fujita & Yoshida, 2024). Before we look at how the two assumptions enable the cue-based memory retrieval hypothesis to predict the grammatical asymmetry, let us establish their validity through empirical evidence.^9^

The assumption of the heavily weighted structure-based cue is supported by research showing that ungrammatical sentences with a feature-matching distractor are analysed as ungrammatical at rates above 0.5. This trend persists even in time-pressured tasks where error-prone responses are expected (e.g., Fujita & Cunnings, 2022; González Alonso et al., 2021; Hammerly et al., 2019; Lago et al., 2019; Parker, 2019b; Schlueter et al., 2018; Tanner et al., 2014). This body of research suggests that during the initial retrieval phase, the parser retrieves the structurally accessible target representation rather than the distractor.

Of particular importance, Schlueter et al. (2019) provided compelling evidence in support of the first assumption. In their study, participants read sentences word by word, such as “[_NP1_ The boy by [_NP2_ the trees]] are really very CHUBBY/GREEN”. At the end of each sentence, they had to choose one of the adjectives that would continue the sentence. These adjectives corresponded semantically to either NP1 (the boy…CHUBBY) or NP2 (the trees…GREEN). The activation-based and multiplicative cue-based models predict that the retrieval probability of NP1 at the copula verb should be around 0.5. Consequently, according to these models, participants should choose the adjective that semantically matches NP1 with a probability of around 0.5. However, Schlueter et al. reported that the retrieval probability of NP1 was around 0.85, suggesting that the structure-based cue is heavily weighted.^10^

The assumption that the structure-based cue is heavily weighted is also supported by findings that interference effects sometimes show a delay relative to mismatch effects (e.g., Fujita & Cunnings, 2024; Fujita & Yoshida, 2024; Jäger et al., 2020; Parker & Phillips, 2017; Sturt, 2003). These findings potentially suggest that initial memory retrieval relies heavily on structural information, with other cues becoming more influential as the revision process begins.

Let us now explore how the two assumptions allow the cue-based hypothesis to predict the grammatical asymmetry. This exploration is based on the activation-based model. Recall that in this model, the spreading activation is calculated as follows: ${A}_i=\sum_j{W}_j{S}_{ji}$. For grammatical sentences with a feature-matching (G-FM) or feature-mismatching (G-FMM) distractor, different associative strengths are calculated between *j* and *i*. Specifically, for the G-FM sentence, *S*_*str*, *NP*1_ = 1 and *S*_*num*, *NP*1_ ≈ .31, and for the G-FMM sentence, *S*_*str*, *NP*1_ = 1, *S*_*num*, *NP*1_ = 1 (feature match = 1, feature mismatch = 0, m = 1, str = structure-based cue, num = number-based cue). With these values, the spreading activation to NP1 is calculated as: *A*_*NP*1_ = .5 · 1 + .5 · .31 ≈ .65 for the G-FM sentence and *A*_*NP*1_ = .5 · 1 + .5 · 1 = 1 for the G-FMM sentence, resulting in inhibitory interference. When the structure-based cue is heavily weighted (.999) relative to the number cue (.001), similar spreading activations are obtained between the G-FM sentence (*A*_*NP*1_ = .999 · 1 + .001 · .31 ≈ 1) and the G-FMM sentence *A*_*NP*1_ = .999 · 1 + .001 · 1 = 1.

Importantly, this calculation applies to grammatical sentences with a floating quantifier, even though the parser is unlikely to predict a floating quantifier during real-time sentence processing. The reason for this is that the appearance of a floating quantifier does not trigger a revision process according to the definitions of revision and dependencies given earlier in this paper. Specifically, when a floating quantifier appears during sentence processing, the parser must assign an additional structure, and the trace or copy of NP1 must be adjoined under this structure. However, no existing dependencies are dissociated during the processing of the floating quantifier, i.e., [_TP_ [_NP1t_ The boys who [_NP2_ the girls] saw] [_VP_ t [V_[+plural]_]]] → [_TP_ [_NP1t_ The boys who [_NP2_ the girls] saw] [_VP_ [_QP_ all t] [V_[+plural]_]]].

Now consider ungrammatical sentences with a feature-matching (UG-FM) or feature-mismatching (UG-FMM) distractor. As discussed earlier, these sentences trigger a revision process, and during this process, cue-based memory retrieval is invoked. During the initial retrieval phase, the spreading activation to NP2 is close to zero due to the structure-based cue being heavily weighted. When the revision process occurs, however, other cues are weighted. Focusing on the number cue, this leads to *S*_*numNP*2_ = 1 for the UG-FM sentence and *S*_*num*, *NP*2_ = 0 for the UG-FMM sentence. All else being equal, these different values lead to shorter retrieval times for the UG-FM sentence compared to the UG-FMM sentence (i.e., facilitatory interference).

The two assumptions enable the cue-based hypothesis to predict mismatch effects and the grammatical asymmetry in a natural way and are consistent with the results of the present study. To implement these assumptions computationally, *the Revision Integrated Cue-Based model* (the RICB model) was developed on the basis of the ACT–R architecture. Specific processing times generated by the activation-based model (Lewis & Vasishth, 2005; Vasishth & Engelmann, 2021) and the RICB model were simulated in R. The computational architectures of both models and the simulations are available at https://osf.io/nhc2p/.

In terms of model parameters, with the exception of cue weights and parameters for the revision process, the RICB model adopted mostly the same values as the activation-based model (Vasishth & Engelmann, 2021; see https://osf.io/nhc2p/). It is important to note that the activation-based model relies on stochastic noise to predict facilitatory interference, whereas the RICB model does not. Nevertheless, the noise component was retained in the RICB model for the purpose of model comparison. The two models were compared by simulation. Each model was run for 10,000 iterations, i.e., 10,000 data points for each of the G-FM, G-FMM, UG-FM and UG-FMM conditions.

**Fig. 9:**
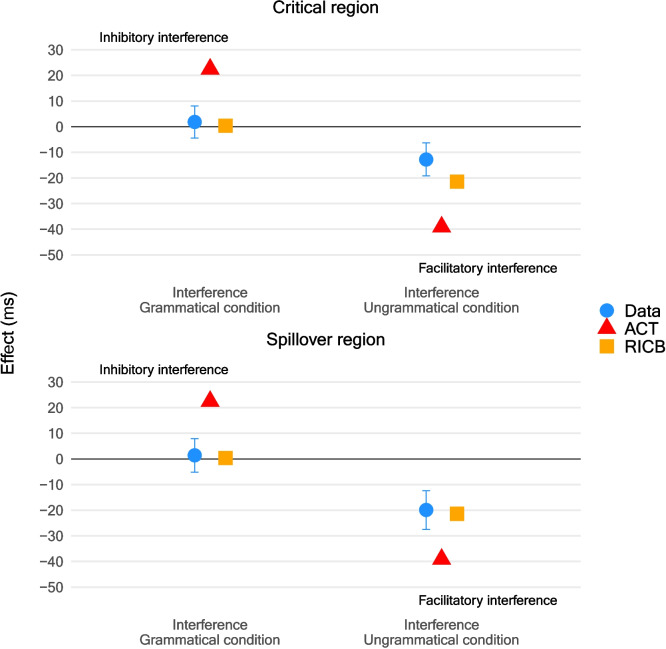
The effects of similarity-based interference derived from the activation-based model, the RICB model, and the analysis of the pooled data reported in the present study

To assess the performance of the two models, the mean absolute errors (MAEs) were calculated: $MAE=\frac{\sum_i\left|{x}_i\hbox{--} {y}_i\right|}{n}$. These MAEs represent the mean differences between the interference effects generated by the models (*y*) and those observed at the critical and spillover regions in the present study (*x*). The simulations showed that the RICB model produced MAEs of 5.01 at the critical region and 1.01 at the spillover region. The activation-based model produced higher MAEs of 23.3 at the critical region and 20.3 at the spillover region, indicating that the RICB model provides a better fit to the observed data. The mean interference effects from these simulations are illustrated in Fig. 9.

The simulations suggest that the RICB model successfully predicts the observed data. However, it raises questions about previous research that has reported inhibitory interference or an opposite interference effect in grammatical sentences. While these discrepancies may be due to a Type I error, the interference effect in grammatical sentences could arise from two potential sources.

One is the time-dependent decay of target representations. Given that real-time sentence processing is constrained by limited memory capacity, encoded representations in working memory should gradually lose their robustness over time, which could lead to interference effects even within grammatical sentences.

The other source, linked to this time-dependent decay, is related to how the human parser operates in noisy environments. These disturbances can affect the perception and processing of input, either through internal factors (e.g., shifts in attention) or external influences (e.g., background noise).

These interrelated factors may lead to interference effects in grammatical sentences. If this hypothesis holds, interference effects in grammatical sentences should be more likely to be observed in lengthy experiments (e.g., too many experimental/filler materials), in experiments with limited statistical power, or in sentences that are difficult to parse due to structural complexity, heavily long-distance dependencies, or the presence of multiple distractors. Some previous studies have observed interference effects in grammatical sentences under such conditions (e.g., Nicenboim et al., 2018; Van Dyke, 2007). This hypothesis could explain the relatively small size and inconsistent occurrence of interference effects in grammatical sentences.

Given the grammatical asymmetry observed in Experiments 1 and 2, this paper suggests that the human parsing system may be inherently insusceptible to interference effects in grammatical sentences. However, performance level factors may be the driving force behind such effects.

## Conclusion

This study investigated memory retrieval during real-time sentence processing. With two empirical experiments testing similarity-based interference in the quantifier float construction, computational modelling and simulations, the primary aims were twofold: First, to assess the predictive capabilities of existing cue-based models of memory retrieval, and second, to test several related hypotheses. The results showed that memory retrieval for floating quantifiers relies on structural information. Similarity-based interference was observed in ungrammatical sentences but not in grammatical sentences, a finding that challenges existing models of cue-based memory retrieval. To reconcile this grammatical asymmetry within the framework of cue-based memory retrieval, this paper proposes that the structure-based cue is heavily weighted and incorporates the concepts of initial memory retrieval and revision. A computational model embodying these assumptions was presented and evaluated through simulations. The results of these simulations showed that the proposed model successfully predicted the observed data.

## Data Availability

Data, analysis code, experimental materials, computational models, and simulations are available at https://osf.io/nhc2p/.

## Footnotes

1 These syntactic structures are based on *X-bar theory* (Chomsky, 1970, 1981, 1995; Jackendoff, 1977; Kornai & Pullum, 1990; Stowell, 1981). For an introduction to X-bar theory, see Haegeman (1994), Hornstein et al. (2005), Lasnik and Uriagereka (1988), and van Riemsdijk and Williams (1986)

2 To streamline the presentation and improve readability, some bar levels are omitted.

3 For expository purposes, this paper focuses on the structure-based cue and the number-based cue for subject-verb agreement and quantifier float.

4 One problem with this assumption is that in ungrammatical sentences, the memory retrieval process is never complete. To address this, I assume that the parser only performs a single memory retrieval after recognising that there is no grammatical dependency.

5 The term “revision” is based on studies of locally ambiguous sentences. The use of this term is partly inspired by Wagers et al. (2009), who hypothesise that mismatch effects initiate a revision process. Whether the search process should be considered a form of revision is an interesting research question.

6 Node *a* dominates node *b* if *a* occupies a higher position in a tree than *b* and the trace from *a* to *b* follows a strictly downward trajectory. Nodes *a* and *b* are sisters if node *c* immediately dominates *a* and *b* and *a* ≠ *b*.

7 Inhibitory interference is based on the activation-based model. Wagers et al. (2009) argue that cue-based memory retrieval, triggered by prediction errors, involves the occasional retrieval of a feature-matching distractor. If we extrapolate this idea to grammatical sentences with a floating quantifier, it means that the parser occasionally retrieves NP2 that matches the quantifier in number. This retrieval violates grammatical constraints and is therefore expected to lead to increased reading times due to revision.

8 One possibility is that NP1, which is held in memory for subject-verb dependency formation (Chen et al., 2005; Gibson, 1998; Kim et al., 2020), contains information about structural relations (e.g., c-command). Given that subject-verb agreement is generally established within a local c-command domain, this is a possible scenario.

9 The editor asked whether a different approach could predict the current results. The discussion here is based on the feature-based direct access mechanism, where the cue-based model technically predicts that hierarchical syntactic structures are not represented as a tree but are encoded as bundles of features. An alternative approach to dependency formation is an iterative search algorithm, where hierarchical syntactic structures are actually represented, and the parser searches through the path of nodes in the syntactic structures to form dependencies. This algorithm is not supported in the language comprehension literature, as some previous work suggests that the parser uses a direct access mechanism during dependency formation (see Kush, 2013; Martin & McElree, 2009; McElree, 2000; McElree et al., 2003). However, it would not be impossible to use phrase structure trees to directly access specific elements for dependency formation, given that phrase structure trees are data structures. In this approach, it can be assumed that the parser first retrieves the structurally permissible element during dependency formation using syntactic structures, and if this element violates the linguistic agreement, the parser searches for an alternative element that licenses it in terms of lexical features.

10 The results of Schlueter et al. also suggest that during the revision process, although other retrieval cues are weighted to search for an alternative element to license the agreement in terms of lexical features, the structurally accessible element remains to be analysed as related to the predicate.
