# Correction to: Memory Retrieval in Online Sentence Parsing: Empirical Evidence, Computational Modelling, and Simulations

**DOI:** 10.1007/s42113-024-00215-7

**Published:** 2024-08-01

**Authors:** Hiroki Fujita

**Affiliations:** Department of Linguistics, University of Potsdam, Karl-Liebknecht-Straße 24–25, 14476 Potsdam, Germany

**Correction to: Computational Brain & Behavior**

https://doi.org/10.1007/s42113-024-00206-8

The original online version of this article was revised to correct the label for Experiment 1, experimental sentence 6b, as follows:

(6b) Grammatical, Distractor mismatch

The article was also revised to correct the label for Experiment 2, experimental sentence 7b, as follows:

(7b) Grammatical, Distractor mismatch

The original article has been corrected.

